# Ethyl 3-methyl-2,6-diphenyl­piperidine-1-carboxyl­ate

**DOI:** 10.1107/S1600536811019155

**Published:** 2011-05-25

**Authors:** Sampath Natarajan, Rita Mathews

**Affiliations:** a Department of Advanced Technology Fusion, Konkuk University, 1 Hwayang-dong, Gwangjin-gu, Seoul-143 701, Republic of Korea

## Abstract

In the title compound, C_21_H_25_NO_2_, the piperidine ring adopts a twisted boat conformation characterized by puckering parameters θ = 89.5 (1) and ϕ = 257.5 (2)°. The phenyl groups are located in equatorial and axial positions on the central piperidine ring, while the methyl group is in an equatorial position. The dihedral angle between the phenyl rings is 49.8 (1)°. An intra­molecular C—H⋯O inter­action occurs. The crystal structure features weak inter­molecular C—H⋯O inter­actions and a stabilizing inter­molecular C—H⋯π contact involving the axial phenyl ring.

## Related literature

For the biological activity of related piperidines, see: Parthiban *et al.* (2009 ▶); Aridoss *et al.* (2007 ▶). For ring conformational analysis, see: Cremer & Pople (1975 ▶); Nardelli (1995 ▶). For the conformation of piperidine derivatives, see: Ravindran *et al.* (1991 ▶); Krishna Kumar & Krishna Pillay (1996 ▶). For the synthesis of the title compound, see: Sampath *et al.* (2003 ▶); Noller & Baliah (1948 ▶).
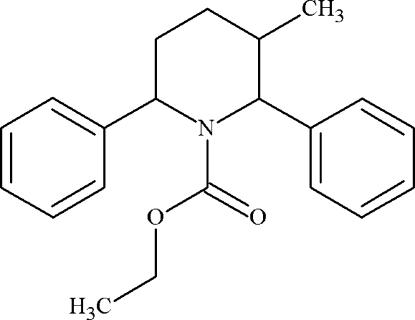


## Experimental

### 

#### Crystal data

C_21_H_25_NO_2_
*M* *_r_* = 323.42
Monoclinic, 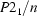

*a* = 10.4113 (3) Å
*b* = 10.6073 (6) Å
*c* = 16.2782 (6) Å
β = 95.960 (2)°
*V* = 1787.98 (13) Å^3^
*Z* = 4
Cu *K*α radiation
μ = 0.60 mm^−1^
*T* = 293 K
0.26 × 0.22 × 0.18 mm

#### Data collection

Enraf–Nonius CAD-4 diffractometer
3698 measured reflections
3502 independent reflections
2428 reflections with *I* > 2σ(*I*)
*R*_int_ = 0.012
Standard reflections: 3; every 60 minutes  intensity decay: none

#### Refinement

*R*[*F* ^2^ > 2σ(*F* ^2^)] = 0.045
*wR*(*F* ^2^) = 0.136
*S* = 1.03
3502 reflections
218 parameters
H-atom parameters constrained
Δρ_max_ = 0.17 e Å^−3^
Δρ_min_ = −0.20 e Å^−3^

### 

Data collection: *CAD-4 EXPRESS* (Enraf–Nonius, 1994 ▶); cell refinement: *CAD-4 EXPRESS*; data reduction: *XCAD4* (Harms, 1996 ▶); program(s) used to solve structure: *SHELXS97* (Sheldrick, 2008 ▶); program(s) used to refine structure: *SHELXL97* (Sheldrick, 2008 ▶); molecular graphics: *ORTEP-3* (Farrugia, 1997 ▶) and *PLATON* (Spek, 2009 ▶); software used to prepare material for publication: *SHELXL97* and *PLATON*.

## Supplementary Material

Crystal structure: contains datablocks I, global. DOI: 10.1107/S1600536811019155/bh2353sup1.cif

Structure factors: contains datablocks I. DOI: 10.1107/S1600536811019155/bh2353Isup2.hkl

Supplementary material file. DOI: 10.1107/S1600536811019155/bh2353Isup3.cml

Additional supplementary materials:  crystallographic information; 3D view; checkCIF report

## Figures and Tables

**Table 1:** Hydrogen-bond geometry (Å, °) *Cg*1 is the centroid of the C13–C18 phenyl ring.

*D*—H⋯*A*	*D*—H	H⋯*A*	*D*⋯*A*	*D*—H⋯*A*
C18—H18⋯O1	0.93	2.91	3.519 (2)	125
C14—H14⋯O2^i^	0.93	2.82	3.406 (2)	122
C10—H10⋯O1^ii^	0.93	2.67	3.460 (3)	144
C3—H3*B*⋯*Cg*1^iii^	0.97	2.70	3.666 (2)	172

Symmetry codes: (i) 
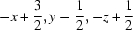
; (ii) 
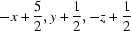
; (iii) 
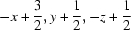
.

**Fig. 1.**
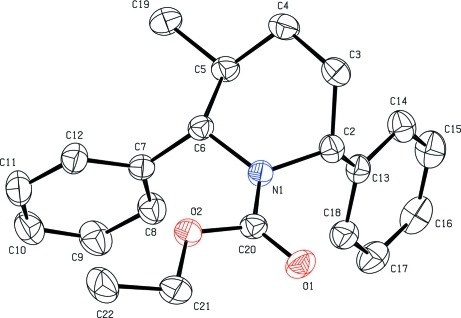
ORTEP diagram of the title molecule with displacement ellipsoid drawn at the 30% probability level. Hydrogen atoms were removed for clarity.

**Fig. 2.**
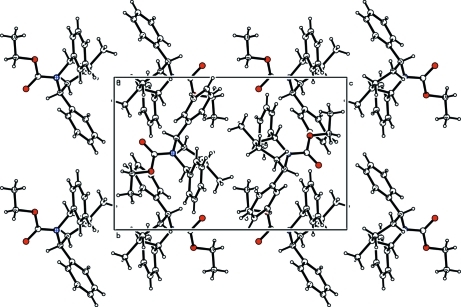
A unit cell packing of the crystal structure of the title compound viewed down a-axis.

## supplementary crystallographic information

### Comment

Piperidines and its *N*-substituted derivatives show significant pharmacological properties (Parthiban *et al.*, 2009; Aridoss *et al.*, 2007). Substitution by electron withdrawing groups (CHO, COCH_2_CH_3_, COPh, NO, *etc*.) at *N*-th position of piperidine ring causes major changes in the ring conformation (Ravindran *et al.*, 1991; Krishna Kumar & Krishna Pillay, 1996). In the title compound (Fig. 1), the ethylacetate group substituting the piperidine ring shows extended conformation and the hetero π electron delocalization through the atoms N1, C20, O1 and O2 causes twisted boat conformation for the piperidine core, with puckering amplitude, Q_T_ = 0.718 (1) Å and phase angle = 89.5 (2)° (Nardelli, 1995; Cremer & Pople, 1975).

The phenyl rings at C2 and C6 atoms are oriented in the axial and equatorial positions, respectively, and the dihedral angle between them is 49.8 (1)°. Similarly, the methyl group at C5 is also oriented in equatorial position. All these substitutions are confirmed by the respective torsion angles. In addition, the substitution of ethylacetate group on N1 atom showed extended conformation with respect to the piperidine ring, which is also confirmed by the torsion angles.

The packing diagram of the title compound viewed down *a*-axis is shown in Fig. 2. The molecules did not present any classical H-bonds. However, the molecules are involved in weak intra- and intermolecular C—H···O interactions (Table 1), which stabilize the molecules in the crystal packing. Interestingly, a C—H···π interaction (C3—H3B···*Cg*1; *Cg*1 is the centroid of the ring C13···C18) also helps for the crystal packing.

### Experimental

The compound, 3-methyl-2,6-diphenylpiperidin-4-one was obtained by adopting an earlier method (Sampath *et al.*, 2003; Noller & Baliah, 1948) and it was reduced using amalgamated zinc in aqueous methanol solution in the presence of HCl, giving 3-methyl-2,6-diphenylpiperidine as a product. To a well stirred solution of 3,5-dimethyl-2,6-diphenylpiperidin-4-one (2 m*M*) and triethylamine (4 m*M*) in freshly distilled benzene (50 ml), a little excess amount of ethylchloroacetate (2.2 m*M*) in benzene (10 ml) was added drop-wise over about half an hour and stirring was continued until the completion of reaction. The reaction mixture was then poured into water and extracted with dichloromethane. Recrystallization of the title compound using pure ethanol resulted in suitable colorless crystals.

### Refinement

H atoms were positioned geometrically and refined using a riding model with C—H = 0.93 Å for aromatic H, 0.97 Å for methylene, 0.98 Å for methine, and 0.96 Å for methyl H atoms. The *U*_iso_ parameters for H atoms were constrained to be 1.5*U*_eq_ of the carrier atom for the methyl H atoms and 1.2*U*_eq_ of the carrier atom for the remaining H atoms.

### Crystal data

**Table:**

C_21_H_25_NO_2_	*F*(000) = 696
*M**_r_* = 323.42	*D*_x_ = 1.201 Mg m^−^^3^
Monoclinic, *P*2_1_/*n*	Cu *K*α radiation, λ = 1.54178 Å
Hall symbol: -P 2yn	Cell parameters from 25 reflections
*a* = 10.4113 (3) Å	θ = 0–90°
*b* = 10.6073 (6) Å	µ = 0.60 mm^−^^1^
*c* = 16.2782 (6) Å	*T* = 293 K
β = 95.960 (2)°	Needle, colourless
*V* = 1787.98 (13) Å^3^	0.26 × 0.22 × 0.18 mm
*Z* = 4	

### Data collection

**Table:**

Enraf–Nonius CAD-4 diffractometer	*R*_int_ = 0.012
Radiation source: fine-focus sealed tube	θ_max_ = 72.0°, θ_min_ = 4.8°
graphite	*h* = 0→12
ω scans	*k* = 0→13
3698 measured reflections	*l* = −20→19
3502 independent reflections	3 standard reflections every 60 min
2428 reflections with *I* > 2σ(*I*)	intensity decay: none

### Refinement

**Table:**

Refinement on *F*^2^	Secondary atom site location: difference Fourier map
Least-squares matrix: full	Hydrogen site location: inferred from neighbouring sites
*R*[*F*^2^ > 2σ(*F*^2^)] = 0.045	H-atom parameters constrained
*wR*(*F*^2^) = 0.136	*w* = 1/[σ^2^(*F*_o_^2^) + (0.0736*P*)^2^ + 0.2748*P*] where *P* = (*F*_o_^2^ + 2*F*_c_^2^)/3
*S* = 1.03	(Δ/σ)_max_ = 0.001
3502 reflections	Δρ_max_ = 0.17 e Å^−^^3^
218 parameters	Δρ_min_ = −0.20 e Å^−^^3^
0 restraints	Extinction correction: *SHELXL97* (Sheldrick, 2008), Fc^*^=kFc[1+0.001xFc^2^λ^3^/sin(2θ)]^-1/4^
0 constraints	Extinction coefficient: 0.0022 (4)
Primary atom site location: structure-invariant direct methods	

### Fractional atomic coordinates and isotropic or equivalent isotropic displacement parameters (Å^2^)

**Table:**

	*x*	*y*	*z*	*U*_iso_*/*U*_eq_	
O1	0.92030 (13)	0.42372 (12)	0.12110 (7)	0.0586 (4)	
O2	0.99447 (12)	0.61660 (11)	0.15872 (7)	0.0516 (3)	
N1	0.90776 (13)	0.49725 (13)	0.25183 (8)	0.0427 (3)	
C2	0.81474 (15)	0.39802 (16)	0.26812 (10)	0.0455 (4)	
H2	0.7841	0.3620	0.2142	0.055*	
C3	0.69751 (17)	0.45929 (18)	0.30030 (11)	0.0516 (4)	
H3A	0.6370	0.3939	0.3123	0.062*	
H3B	0.6550	0.5127	0.2574	0.062*	
C4	0.73118 (18)	0.5382 (2)	0.37787 (12)	0.0584 (5)	
H4A	0.7073	0.4918	0.4254	0.070*	
H4B	0.6810	0.6153	0.3735	0.070*	
C5	0.87457 (16)	0.57150 (17)	0.39184 (10)	0.0470 (4)	
H5	0.9212	0.4975	0.4155	0.056*	
C6	0.92925 (15)	0.60452 (16)	0.30998 (9)	0.0414 (4)	
H6	0.8820	0.6777	0.2857	0.050*	
C7	1.07053 (16)	0.63999 (16)	0.32679 (9)	0.0435 (4)	
C8	1.16351 (18)	0.55226 (19)	0.35314 (13)	0.0587 (5)	
H8	1.1416	0.4675	0.3555	0.070*	
C9	1.2899 (2)	0.5901 (2)	0.37612 (15)	0.0718 (6)	
H9	1.3516	0.5301	0.3942	0.086*	
C10	1.3249 (2)	0.7134 (2)	0.37269 (14)	0.0680 (6)	
H10	1.4095	0.7378	0.3888	0.082*	
C11	1.2344 (2)	0.8007 (2)	0.34532 (14)	0.0686 (6)	
H11	1.2576	0.8851	0.3421	0.082*	
C12	1.10831 (19)	0.76434 (18)	0.32231 (12)	0.0563 (5)	
H12	1.0477	0.8249	0.3034	0.068*	
C13	0.87742 (17)	0.28998 (16)	0.31910 (10)	0.0459 (4)	
C14	0.81423 (19)	0.22492 (19)	0.37698 (11)	0.0565 (5)	
H14	0.7339	0.2528	0.3897	0.068*	
C15	0.8691 (2)	0.1193 (2)	0.41592 (12)	0.0676 (6)	
H15	0.8256	0.0768	0.4546	0.081*	
C16	0.9876 (2)	0.0766 (2)	0.39778 (13)	0.0685 (6)	
H16	1.0241	0.0051	0.4237	0.082*	
C17	1.0513 (2)	0.1403 (2)	0.34120 (13)	0.0657 (5)	
H17	1.1318	0.1121	0.3291	0.079*	
C18	0.99719 (19)	0.24578 (18)	0.30200 (12)	0.0558 (5)	
H18	1.0416	0.2879	0.2636	0.067*	
C19	0.8953 (2)	0.6789 (2)	0.45370 (11)	0.0642 (5)	
H19A	0.9856	0.6992	0.4622	0.096*	
H19B	0.8659	0.6536	0.5052	0.096*	
H19C	0.8476	0.7515	0.4328	0.096*	
C20	0.93902 (16)	0.50599 (16)	0.17288 (10)	0.0440 (4)	
C21	1.0362 (2)	0.6314 (2)	0.07700 (12)	0.0637 (5)	
H21A	0.9644	0.6169	0.0351	0.076*	
H21B	1.1039	0.5713	0.0688	0.076*	
C22	1.0848 (3)	0.7612 (2)	0.07088 (15)	0.0802 (7)	
H22A	1.1132	0.7740	0.0172	0.120*	
H22B	1.1558	0.7744	0.1125	0.120*	
H22C	1.0169	0.8199	0.0789	0.120*	

### Atomic displacement parameters (Å^2^)

**Table:**

	*U*^11^	*U*^22^	*U*^33^	*U*^12^	*U*^13^	*U*^23^
O1	0.0793 (9)	0.0536 (8)	0.0446 (7)	−0.0032 (7)	0.0148 (6)	−0.0103 (6)
O2	0.0663 (8)	0.0519 (7)	0.0388 (6)	−0.0084 (6)	0.0165 (5)	0.0009 (5)
N1	0.0506 (8)	0.0425 (7)	0.0355 (7)	−0.0050 (6)	0.0076 (6)	−0.0007 (6)
C2	0.0476 (9)	0.0477 (9)	0.0408 (8)	−0.0065 (7)	0.0024 (7)	0.0012 (7)
C3	0.0453 (9)	0.0592 (11)	0.0500 (10)	−0.0015 (8)	0.0038 (7)	0.0055 (8)
C4	0.0522 (10)	0.0690 (13)	0.0566 (11)	−0.0021 (9)	0.0179 (8)	−0.0046 (9)
C5	0.0513 (9)	0.0530 (10)	0.0378 (8)	−0.0001 (8)	0.0095 (7)	−0.0017 (7)
C6	0.0464 (9)	0.0409 (8)	0.0376 (8)	0.0002 (7)	0.0068 (6)	−0.0019 (6)
C7	0.0476 (9)	0.0451 (9)	0.0386 (8)	−0.0019 (7)	0.0077 (7)	−0.0015 (7)
C8	0.0526 (11)	0.0519 (11)	0.0712 (12)	−0.0010 (9)	0.0048 (9)	0.0060 (9)
C9	0.0508 (11)	0.0749 (15)	0.0881 (16)	0.0031 (10)	0.0001 (10)	0.0103 (12)
C10	0.0519 (11)	0.0813 (15)	0.0705 (13)	−0.0152 (11)	0.0052 (9)	−0.0012 (11)
C11	0.0689 (13)	0.0583 (12)	0.0788 (14)	−0.0205 (11)	0.0080 (11)	−0.0033 (11)
C12	0.0594 (11)	0.0460 (10)	0.0630 (11)	−0.0039 (9)	0.0044 (9)	−0.0018 (8)
C13	0.0528 (9)	0.0429 (9)	0.0414 (8)	−0.0076 (7)	0.0023 (7)	−0.0010 (7)
C14	0.0594 (11)	0.0592 (11)	0.0507 (10)	−0.0094 (9)	0.0047 (8)	0.0073 (9)
C15	0.0900 (16)	0.0600 (12)	0.0514 (11)	−0.0104 (11)	0.0015 (10)	0.0136 (9)
C16	0.0953 (16)	0.0522 (12)	0.0539 (11)	0.0051 (11)	−0.0124 (11)	0.0025 (9)
C17	0.0695 (13)	0.0556 (12)	0.0705 (13)	0.0114 (10)	0.0005 (10)	−0.0046 (10)
C18	0.0613 (11)	0.0481 (10)	0.0591 (10)	0.0012 (9)	0.0116 (9)	−0.0002 (8)
C19	0.0748 (13)	0.0732 (14)	0.0466 (10)	−0.0042 (11)	0.0162 (9)	−0.0146 (9)
C20	0.0492 (9)	0.0448 (9)	0.0389 (8)	0.0021 (7)	0.0094 (7)	0.0016 (7)
C21	0.0800 (13)	0.0707 (13)	0.0445 (10)	−0.0055 (11)	0.0266 (9)	0.0037 (9)
C22	0.0898 (16)	0.0844 (16)	0.0706 (13)	−0.0249 (13)	0.0288 (12)	0.0115 (12)

### Geometric parameters (Å, °)

**Table:**

O1—C20	1.2148 (19)	C10—C11	1.362 (3)
O2—C20	1.338 (2)	C10—H10	0.9300
O2—C21	1.4500 (19)	C11—C12	1.382 (3)
N1—C20	1.3612 (19)	C11—H11	0.9300
N1—C2	1.473 (2)	C12—H12	0.9300
N1—C6	1.482 (2)	C13—C18	1.387 (2)
C2—C13	1.522 (2)	C13—C14	1.388 (2)
C2—C3	1.523 (2)	C14—C15	1.382 (3)
C2—H2	0.9800	C14—H14	0.9300
C3—C4	1.525 (3)	C15—C16	1.375 (3)
C3—H3A	0.9700	C15—H15	0.9300
C3—H3B	0.9700	C16—C17	1.368 (3)
C4—C5	1.528 (2)	C16—H16	0.9300
C4—H4A	0.9700	C17—C18	1.379 (3)
C4—H4B	0.9700	C17—H17	0.9300
C5—C19	1.521 (2)	C18—H18	0.9300
C5—C6	1.543 (2)	C19—H19A	0.9600
C5—H5	0.9800	C19—H19B	0.9600
C6—C7	1.515 (2)	C19—H19C	0.9600
C6—H6	0.9800	C21—C22	1.474 (3)
C7—C8	1.379 (2)	C21—H21A	0.9700
C7—C12	1.381 (2)	C21—H21B	0.9700
C8—C9	1.389 (3)	C22—H22A	0.9600
C8—H8	0.9300	C22—H22B	0.9600
C9—C10	1.361 (3)	C22—H22C	0.9600
C9—H9	0.9300		
			
C20—O2—C21	115.37 (14)	C10—C11—C12	120.3 (2)
C20—N1—C2	116.41 (13)	C10—C11—H11	119.9
C20—N1—C6	121.04 (13)	C12—C11—H11	119.9
C2—N1—C6	119.48 (12)	C7—C12—C11	121.33 (19)
N1—C2—C13	112.56 (13)	C7—C12—H12	119.3
N1—C2—C3	108.81 (14)	C11—C12—H12	119.3
C13—C2—C3	116.58 (14)	C18—C13—C14	117.88 (17)
N1—C2—H2	106.0	C18—C13—C2	119.25 (15)
C13—C2—H2	106.0	C14—C13—C2	122.56 (16)
C3—C2—H2	106.0	C15—C14—C13	120.83 (19)
C2—C3—C4	113.29 (15)	C15—C14—H14	119.6
C2—C3—H3A	108.9	C13—C14—H14	119.6
C4—C3—H3A	108.9	C16—C15—C14	120.4 (2)
C2—C3—H3B	108.9	C16—C15—H15	119.8
C4—C3—H3B	108.9	C14—C15—H15	119.8
H3A—C3—H3B	107.7	C17—C16—C15	119.4 (2)
C3—C4—C5	112.78 (14)	C17—C16—H16	120.3
C3—C4—H4A	109.0	C15—C16—H16	120.3
C5—C4—H4A	109.0	C16—C17—C18	120.6 (2)
C3—C4—H4B	109.0	C16—C17—H17	119.7
C5—C4—H4B	109.0	C18—C17—H17	119.7
H4A—C4—H4B	107.8	C17—C18—C13	120.92 (19)
C19—C5—C4	109.96 (15)	C17—C18—H18	119.5
C19—C5—C6	111.23 (15)	C13—C18—H18	119.5
C4—C5—C6	111.50 (14)	C5—C19—H19A	109.5
C19—C5—H5	108.0	C5—C19—H19B	109.5
C4—C5—H5	108.0	H19A—C19—H19B	109.5
C6—C5—H5	108.0	C5—C19—H19C	109.5
N1—C6—C7	112.66 (13)	H19A—C19—H19C	109.5
N1—C6—C5	109.43 (13)	H19B—C19—H19C	109.5
C7—C6—C5	109.80 (13)	O1—C20—O2	123.46 (15)
N1—C6—H6	108.3	O1—C20—N1	124.70 (16)
C7—C6—H6	108.3	O2—C20—N1	111.84 (14)
C5—C6—H6	108.3	O2—C21—C22	107.49 (17)
C8—C7—C12	117.81 (17)	O2—C21—H21A	110.2
C8—C7—C6	121.71 (16)	C22—C21—H21A	110.2
C12—C7—C6	120.28 (16)	O2—C21—H21B	110.2
C7—C8—C9	120.24 (19)	C22—C21—H21B	110.2
C7—C8—H8	119.9	H21A—C21—H21B	108.5
C9—C8—H8	119.9	C21—C22—H22A	109.5
C10—C9—C8	121.1 (2)	C21—C22—H22B	109.5
C10—C9—H9	119.4	H22A—C22—H22B	109.5
C8—C9—H9	119.4	C21—C22—H22C	109.5
C9—C10—C11	119.2 (2)	H22A—C22—H22C	109.5
C9—C10—H10	120.4	H22B—C22—H22C	109.5
C11—C10—H10	120.4		
			
C20—N1—C2—C13	108.65 (16)	C8—C9—C10—C11	−0.8 (4)
C6—N1—C2—C13	−90.94 (17)	C9—C10—C11—C12	0.8 (3)
C20—N1—C2—C3	−120.56 (16)	C8—C7—C12—C11	−1.5 (3)
C6—N1—C2—C3	39.84 (19)	C6—C7—C12—C11	173.46 (16)
N1—C2—C3—C4	−57.33 (19)	C10—C11—C12—C7	0.4 (3)
C13—C2—C3—C4	71.2 (2)	N1—C2—C13—C18	−41.5 (2)
C2—C3—C4—C5	17.3 (2)	C3—C2—C13—C18	−168.23 (16)
C3—C4—C5—C19	163.67 (16)	N1—C2—C13—C14	145.07 (16)
C3—C4—C5—C6	39.8 (2)	C3—C2—C13—C14	18.3 (2)
C20—N1—C6—C7	−62.3 (2)	C18—C13—C14—C15	−0.2 (3)
C2—N1—C6—C7	138.25 (14)	C2—C13—C14—C15	173.29 (17)
C20—N1—C6—C5	175.28 (14)	C13—C14—C15—C16	−0.1 (3)
C2—N1—C6—C5	15.8 (2)	C14—C15—C16—C17	0.5 (3)
C19—C5—C6—N1	179.64 (15)	C15—C16—C17—C18	−0.5 (3)
C4—C5—C6—N1	−57.23 (19)	C16—C17—C18—C13	0.2 (3)
C19—C5—C6—C7	55.50 (19)	C14—C13—C18—C17	0.2 (3)
C4—C5—C6—C7	178.64 (15)	C2—C13—C18—C17	−173.55 (17)
N1—C6—C7—C8	−53.5 (2)	C21—O2—C20—O1	−2.2 (3)
C5—C6—C7—C8	68.8 (2)	C21—O2—C20—N1	177.33 (15)
N1—C6—C7—C12	131.73 (16)	C2—N1—C20—O1	−17.4 (2)
C5—C6—C7—C12	−106.04 (18)	C6—N1—C20—O1	−177.46 (16)
C12—C7—C8—C9	1.6 (3)	C2—N1—C20—O2	163.14 (14)
C6—C7—C8—C9	−173.36 (18)	C6—N1—C20—O2	3.1 (2)
C7—C8—C9—C10	−0.4 (3)	C20—O2—C21—C22	175.60 (17)

### Hydrogen-bond geometry (Å, °)

**Table:**

Cg1 is the centroid of the C13–C18 phenyl ring.

**Table:**

*D*—H···*A*	*D*—H	H···*A*	*D*···*A*	*D*—H···*A*
C2—H2···O1	0.98	2.28	2.749 (2)	109
C18—H18···O1	0.93	2.91	3.519 (2)	125
C14—H14···O2^i^	0.93	2.82	3.406 (2)	122
C10—H10···O1^ii^	0.93	2.67	3.460 (3)	144
C3—H3B···Cg1^iii^	0.97	2.70	3.666 (2)	172

Symmetry codes: (i) −*x*+3/2, *y*−1/2, −*z*+1/2; (ii) −*x*+5/2, *y*+1/2, −*z*+1/2; (iii) −*x*+3/2, *y*+1/2, −*z*+1/2.
